# β-Lactolin Enhances Neural Activity, Indicated by Event-Related P300 Amplitude, in Healthy Adults: A Randomized Controlled Trial

**DOI:** 10.3233/JAD-201413

**Published:** 2021-05-18

**Authors:** Ayana Kanatome, Yasuhisa Ano, Kazushi Shinagawa, Yumiko Ide, Midori Shibata, Satoshi Umeda

**Affiliations:** a Kirin Central Research Institute, Kirin Holdings Company, Ltd., Fujisawa, Kanagawa, Japan; b Department of Psychology, Keio University, Mita, Minato-ku, Tokyo, Japan; c Tokyo Center Clinic, Chuo-ku, Tokyo, Japan

**Keywords:** attention, clinical trial, cognitive function, EEG, β-lactoglobulin, β-lactolin, β-lactopeptide, memory, P300, whey

## Abstract

**Background:**

Epidemiological studies have shown that dairy product consumption is beneficial for cognitive function in elderly individuals. β-lactolin is a Gly–Thr–Trp–Tyr lacto-tetrapeptide rich in fermented dairy products that improves memory retrieval, attention, and executive function in older adults with subjective cognitive decline and prevents the pathology of Alzheimer’s disease in rodents. There has been no study on the effects of β-lactolin on neural activity in humans.

**Objective:**

We investigated the effects of β-lactolin on neural activity and cognitive function in healthy adults.

**Methods:**

In this randomized, double-blind, placebo-controlled study, 30 participants (45–64 years old) consumed β-lactolin or placebo for 6 weeks. Neural activity during auditory and language tasks was measured through 64-channel electroencephalography. Moreover, verbal fluency tests were performed at baseline and after 6 weeks.

**Results:**

The β-lactolin group had a significantly higher P300 amplitude at the Cp2 site (a part of the parietal lobe near the center of brain, *p* = 0.011), and C4 site (the area between the frontal and parietal lobe, *p* = 0.02) during the auditory tasks after 6 weeks than the placebo group. Thus, β-lactolin supplementation promoted neural activity in the parietal area, which increases concentration and attention during auditory cognitive tasks. Compared with the placebo group, the β-lactolin group also showed significant changes in the scores of verbal fluency test after 6 weeks (*p* = 0.033).

**Conclusion:**

Our findings provide insight into the mechanisms underlying the effects of β-lactolin on attention in healthy adults.

## INTRODUCTION

Owing to the rapid increase in the elderly population worldwide, cognitive decline and dementia have become an increasing burden on patients, their families, and national healthcare systems. Additionally, due to the lack of effective therapies for dementia, there has been an increased attention to the preventive approaches. Epidemiological studies have suggested that the consumption of dairy products prevents age-related cognitive decline and reduces the risk of dementia and cognitive decline [1–4]. Interventional studies on rodents and humans have reported that certain dairy products prevent cognitive decline. We previously reported that camembert cheese fermented with *Penicillium* prevents the development of Alzheimer’s disease in a transgenic mouse model. Moreover, we observed that β-lactolin prevented cognitive decline and the pathology of Alzheimer’s disease [5, 6].

β-lactolin is a β-lactoglobulin-derived Gly-Thr-Trp-Tyr tetrapeptide that is rich in dairy products, including camembert cheese, blue cheese fermented using fungi, and whey peptides resulting from whey protein enzymatic digestion [6]. β-lactolin increases monoamine levels in the cortex and hippocampus regions; moreover, it has been shown to improve spatial working memory and attention in mice with pharmacologically induced amnesia [7, 8]. β-lactolin has also been shown to suppress memory impairment and Alzheimer’s disease pathology in an Alzheimer’s disease transgenic mouse model [9]. We previously reported that supplementation with β-lactolin-rich whey peptides for 6 weeks improved the scores of the verbal fluency test (VFT), which evaluates the executive function and memory retrieval, and the Stroop test, which evaluates inhibition of attention, in healthy middle-aged adults compared to those in the placebo group [10]. In healthy older adults, β-lactolin supplementation has been shown to improve selective and sustained attention, as evaluated by the visual-cancellation test, and memory retrieval, as evaluated by paired associative learning, compared to those in the placebo group [11]. Taken together, β-lactolin supplementation improves attention, executive function, and memory retrieval. Therefore, β-lactolin supplementation can prevent cognitive decline and dementia. However, the underlying mechanisms of action of β-lactolin on brain activity in humans remain unclear.

Previous studies have suggested that β-lactolin supplementation improves attention, which is associated with parietal area activation. High temporal resolution measurement is required to evaluate task-related neural activity in the parietal area. Therefore, measurement by electroencephalography (EEG) is suitable for assessing the underlying mechanisms. A previous study reported that supplementation with polyunsaturated fatty acids increases event-related potential (ERP) in the midline-parietal during auditory tasks, which indicates increased neural activity [12]. The P300 wave is an ERP component elicited in the decision-making process, including the evaluation and categorization of stimulus [13, 14]. Its signal is strongly measured by electrodes covering the parietal lobe, and its amplitude correlates with the attention levels for specific stimuli, with a larger P300 amplitude indicating greater attention. Therefore, P300 is considered a concentration and attention indicator. Persons with mild cognitive impairment and Alzheimer’s disease deficits attention and working memory [15, 16]. A meta-analysis revealed that the amplitude of P300 is smaller in subjects with Alzheimer’s disease than in the healthy controls [17]. In particular, it has been suggested that P300 ERP component from auditory stimuli shows small amplitude in Alzheimer’s disease during its early stage [18]. A longitudinal study suggested that the rate of increase in auditory P300 latency was greater in the patients with Alzheimer’s disease than the normal controls [19]. Another group investigating the ERP for evaluating cognitive impairment [20–22] suggested that ERP is associated with the cognitive decline and dementia. The parietal lobe is a part of the association cortex and is involved in the processing of sensory information, perception, decision-making, numerical cognition, integration, speech comprehension, and spatial awareness [23]. Parietal lobe damage causes functional impairment related to spatial perception, including selective attention, directional attention, and depth perception. MRI analysis revealed that the inferior parietal lobe is involved in the monitoring and updating sound location during auditory working tasks [24]. These findings suggest that EEG to obtain ERP measurements, indicative of neural activity in the parietal area, can be used to evaluate the underlying mechanisms of action of β-lactolin on attention. Moreover, previous studies have suggested that β-lactolin supplementation improves memory retrieval. The N400 wave is an ERP component elicited while responding to words and other meaningful (or potentially meaningful) stimuli, including visual and auditory words, language signs, pictures, faces, and environmental sounds [25, 26]. This suggests that evaluating N400 in the frontal area is suitable for evaluating the mechanisms underlying the β-lactolin effects on memory.

We conducted a randomized, double-blind, plac-ebo-controlled study to investigate the effects of β-lactolin on neural activity using ERP measurements obtained through EEG.

## MATERIALS AND METHODS

### Participants

In total, 30 Japanese-speaking healthy adults, aged 45–64 years, who were self-aware of forgetfulness, were enrolled. The participants with suspected dementia, visual or hearing impairment, difficulties in color recognition, anamnesis of epilepsy or cranial nerve disease, depressive symptoms or depressive disorder diagnosis, history of head surgery, hormone treatment, climacterium diagnosis, anamnesis of heart disease or heart pacemakers, anamnesis of febrile seizures, claustrophobia, tinnitus, asthma, hay fever, physical deconditioning due to test foods, diseases requiring regular administration of medicine or anamnesis of severe diseases, treatment for cognitive functions, regular ingestion of drugs or health foods that could affect cognitive function (more than once per week), regular ingestion of foods similar to the test foods or energy drinks (more than once a week), and irregular lifestyles such as shift work were excluded from the study. Additionally, the individuals who were pregnant or nursing, heavy drinkers, and smokers or started smoking cessation within 12 months prior to the study were also excluded. During screening, the inclusion and exclusion criteria were assessed using a questionnaire, Mini-Mental State Examination (MMSE), and interviews by the principal investigator. A previous study on other nutritional ingredients using EEG required 10 to 15 participants per group to detect statistically significant ERP amplitude differences (α= 0.05) [27, 28]. To ensure sufficient statistical power for primary outcomes, we required at least 15 participants per group.

### Statement of ethical approval and registration

The study was conducted in accordance with the principles of Declaration of Helsinki and Ethical Guidelines for Medical and Health Research Involving Human Subjects and was approved by the ethics committee of the Japan Conference of Clinical Research. Written informed consent was obtained from all participants. This study was conducted by a principal investigator of Tokyo Center Clinic. This study was registered on October 01, 2018 in the database of the University Hospital Medical Information Network before enrolling the participants (Registration No. UMIN000034347).

### Experimental supplements

Test tablets containing 1.6 mg of β-lactolin in whey enzymatic digestion were prepared by Kirin Holdings Co., Ltd. (Tokyo, Japan) as described previously [11]. The tablets were ingested daily for 6 weeks at the same time each day. Whey peptides were substituted with a similar amount of dextrin in placebo tablets. The test and placebo tablets were not distinguishable by size, shape, and taste. The amount of β-lactolin was equivalent to that used in the previous studies [10, 11].

### Procedures

**Fig. 1:**
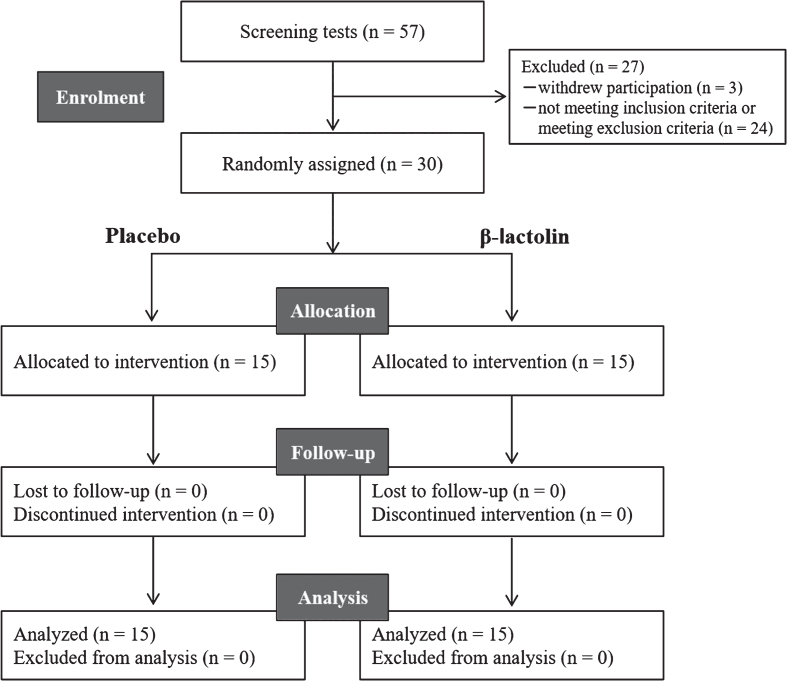
CONSORT diagram. Among the 57 screened participants, 30 were included in this study. Participants were randomly allocated to the placebo (*n* = 15) and β-lactolin (*n* = 15) groups. All participants completed the study and were included in the analysis.

This study employed a randomized, placebo-controlled, double-blind, parallel-group comparative design as described previously [10]. Figure 1 shows the screening procedure. During screening, we assessed the inclusion and exclusion criteria, lifestyle characteristics, MMSE scores, Japanese mild cognitive impairment (MCI) screen scores, the Japanese version of the CNS Vital Signs (Cognitrax) scores, and safety assessments (interview, height, body weight, body mass index, blood pressure, pulse wave, and blood chemical analysis). The selected participants were randomly allocated to the β-lactolin or placebo groups using a computer program to ensure that both the groups were age- and sex-matched. The individual responsible for group allocation was blinded to the eligibility assessments, data collection, and data analysis. Participants, research staff, and outcome assessors were blinded to group allocations until the completion of data analyses. The participants were instructed to maintain regular lifestyles and avoid any drugs, health foods, or protein supplements that could affect cognitive performance during the study period. Compliance was monitored using participation diaries. On the day of neuropsychological tests, the participants were instructed to completely avoid consuming any foods or beverages containing caffeine and to avoid ingesting any foods and beverages except water within 2 h before the start of neuropsychological tests. Neuropsychological tests and ERP measurements were performed at 0 week and after 6 weeks of intervention. The ERP and VFT were recorded at Keio University between October 2018 and July 2019 while the other parameters were recorded at the Tokyo Center Clinic (Tokyo, Japan).

### ERP recording (Primary outcome)

#### Auditory tasks

Three tones at different frequencies (500 Hz, 1 kHz, and 2 kHz) were used to reduce the effect of habituation to the stimulus. Tone presentation was performed at 65 dB using speakers placed approximately 0.75 m from the participants at 45° on either side. A “+” symbol was displayed on the center of the screen, and the participants were instructed to fixate on it. The task involved pressing the space key immediately after the auditory stimuli presentation. The aforementioned tones were randomly presented 90 times using an inter-stimulus interval between 1.5 and 2.5 s to prevent anticipation. This task was performed twice with the approximate total experimental time being 6 min.

#### Lexical decision task

In total, 60 abstract and 60 concrete words were selected from the NTT database based on the lexical properties of the Japanese language, including familiarity, frequency, and accent [29]. Subsequently, they were matched pairwise according to word length and familiarity with the experimental items for concreteness, length, and mean positional bigram frequency. We used the same database to create 120 pseudowords by altering a single letter from each matched word. The procedure was performed as previously described [30]. Stimuli were presented at eye level on a monitor at a distance of approximately 70 cm from the participant. Each trial was conducted as follows: a fixation crosshair was displayed for 700 ms, followed by a blank screen for 300 ms, a string for 300 ms, a blank screen for 300 ms, and finally, a question mark (“word or nonword”) that remained on the screen for 2,500 ms or until a response was provided. Subsequently, another fixation crosshair appeared. The experiment started with 10 practice trials followed by four lexical decision blocks that lasted between 3 min and 5 min each. The stimuli were randomly presented to each participant. To indicate whether the string was a Japanese word, the participants were instructed to position their left or right index fingers on two response buttons marked as word and nonword.

#### Data acquisition

The participants were placed in a dark sound-proofed room during the task and EEG recordings. EEG was recorded during the task using NetStation 5.3.0.1 with a 64-channel HydroCel Geodesic Sensor Net referenced to the vertex (Cz). The sampling rate was 500 Hz. Electrode impedance was maintained at < 50 k*Ω*. Data processing was performed in MATLAB R2018a (MathWorks, Natick, MA, USA) using EEGLAB 14.1.2b [31]. After data acquisition, the continuous EEG signal was filtered with a 0.5–30 Hz band-pass filter and segmented according to the tone onset into 600-ms epochs with a 200-ms baseline period. The raw data underwent auto channel rejection, and powerline fluctuations at 50 Hz were removed using the cleanline EEGLAB plug-in [32]. The data were re-referenced to an average reference. The criterion of artifact rejection was set at +0.0001 V, –0.0001 V. We performed baseline correction for data obtained at 200 ms before the stimulus. P300 and N400 amplitudes were analyzed in the auditory and lexical decision tasks, respectively.

### Neuropsychological tests

In the VFT, which was used to assess executive function and memory retrieval, the participants were asked to verbally name as many items beginning with ‘shi’ and ‘a’ (phonemic fluency) and as many animals as possible (semantic fluency) within one minute. To evaluate amnestic MCI, the memory performance index (MPI) was measured using the Japanese MCI screen, which involves a word recall test per computer instructions [33]. To evaluate total cognitive performance, the neurocognition index (NCI) was measured using Cognitrax, which is a computerized neurocognitive test battery [34]. The NCI was calculated based on the scores of each task and standardized by adjusting the mean score to 100.

### Statistical analysis

Differences in the P300 ERP were assessed using a two-way repeated-measures analysis of variance (ANOVA) with group (β-lactolin, placebo) and time (week 0, week 6) as factors. Differences in the N400 ERP were assessed using three-way ANOVA with group, time, and condition (word, non-word) as factors. The simple effects were analyzed for any statistically significant difference using ANOVA. The statistical analyses were performed using JMP (SAS Institute Japan Ltd., Tokyo, Japan). Between- and within-group comparisons of neuropsychological tests were done using unpaired and paired *t*-test, respectively. This statistical analysis was performed using SAS 9.4 (SAS Institute Japan Ltd.). The statistical significance was set at *p* < 0.05.

## RESULTS

### Baseline characteristics of the study groups

Figure 1 shows a flowchart of the experimental procedure. After screening, 30 participants were included in the study and 27 participants were excluded due to participation withdrawal (*n* = 3), not meeting the inclusion criteria (*n* = 1), visual and hearing impediments (*n* = 1), anamnesis of hay fever (*n* = 1), participation in other clinical trials (*n* = 1), and unsuitability as determined by the principal investigator (*n* = 20). The 30 participants included in the study were randomly allocated to the β-lactolin and placebo groups and received respective supplementation for 6 weeks. No participant withdrew from the study.

As shown in Table 1, there were 15 participants in each group. There were no significant between-group differences in baseline characteristics.

**Table 1:** Baseline characteristics of the participants

Characteristics	Placebo	β-lactolin	*p*
Age (y)	53.0±5.2	53.3±5.2	0.89
Male/Female	7/8	6/9	0.71
Educational history (y)	15.1±1.3	14.7±1.2	0.39
Body weight (kg)	58.45±12.87	57.77±10.25	0.87
Body mass index (kg/m^2^)	21.3±3.42	21.59±2.59	0.80
Systolic blood pressure (mmHg)	123.9±14.3	116.9±11.0	0.15
Diastolic blood pressure (mmHg)	76.5±13.2	77.2±14.8	0.90
Pulse wave (bpm)	76.6±12.0	76.0±8.6	0.88

Data are presented as means±standard deviations for the placebo and β-lactolin groups (*n* = 15 each group); *p*-values were calculated using unpaired *t*-tests, except those for male/female ratios, which were calculated using χ^2^ test.

### ERP results

#### Auditory task

**Fig. 2:**
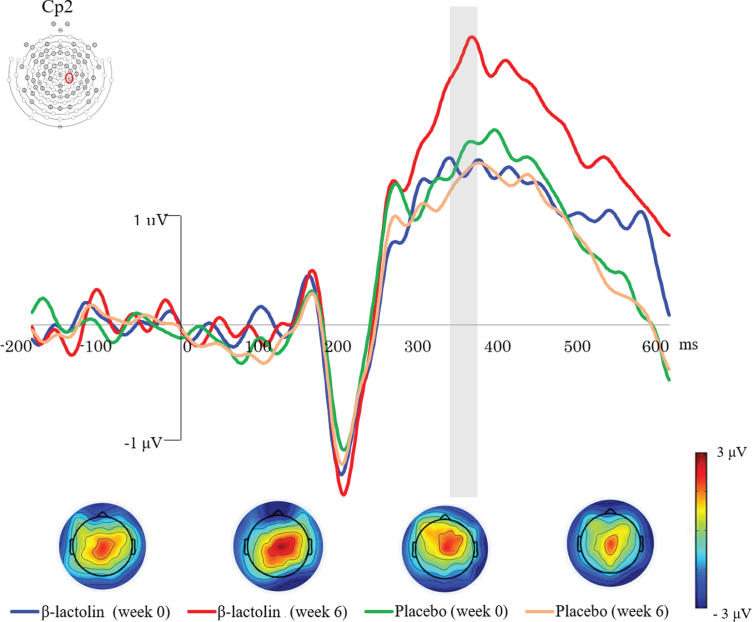
Event-related potential (ERP) waveforms and topographic maps in the auditory tasks. ERP waves for the β-lactolin (blue and red lines) and placebo groups (green and yellow lines) are shown. The topographic maps for P300 (340–360 ms) wave reveal the voltage distribution at different time points (week 0, week 6) for each group (β-lactolin, placebo). Grey rectangle indicates the analyzed time window (340–360 ms).

**Fig. 3:**
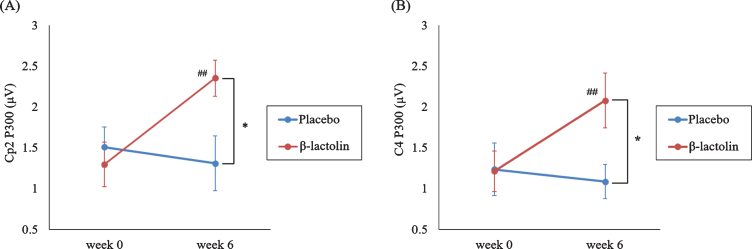
P300 amplitude in the auditory tasks. A, B) P300 amplitude in the central area (C4 site at the area between the frontal and parietal lobe and Cp2 site of a part of the parietal lobe) at different time points (week 0, week 6) for each group (β-lactolin, placebo). Data are presented as means±standard error for the placebo and β-lactolin groups (*n* = 15 each group). ^*^p < 0.05, ^# #^*p* < 0.01.

Based on the previous studies and visual inspection of the grand average waveforms, we used 340–360 ms as the time window for P300 wave. Figure 2 shows the ERP waves in the β-lactolin (blue and red lines) and placebo groups (green and yellow lines). Grey rectangle indicates the analyzed time window (340–360 ms). ANOVA revealed a statistically significant two-way interaction of group (β-lactolin, placebo) and time (week 0, week 6) for P300 at the Cp2 (*F*_ (1,28) _  = 8.11, *p* = 0.008, *η_*p*_*^2^ = 0.23, Fig. 3A) and C4 sites (*F*_ (1,28) _ = 6.02, *p* = 0.021, *η_*p*_*^2^ = 0.18, Fig. 3B). Simple effect tests indicated that the P300 amplitude at week 6 was significantly higher in the β-lactolin group than in the placebo group (at Cp2 site: *t*_ (28) _ = 2.62, *p* = 0.011, *d* = 1.34; at C4 site: *t*_ (28) _ = 2.39, *p* = 0.02, *d* = 1.22). In the β-lactolin group, the P300 amplitude of Cp2 and C4 sites at week 6 was also significantly higher than that at week 0, which was not observed in the placebo group. There was no statistically significant between-group difference at week 0 of the intervention. These results suggested that β-lactolin supplementation promoted neural activity in the parietal area.

#### Lexical decision task

Based on the previous studies and visual inspection of grand average waveforms, we used 300–500 ms as the time window for N400 wave at the Fc2 site. We conducted a three-way (group, time, condition) ANOVA on ERPs at the Fc2 site in this time window. There was a statistically significant effect of condition (nonword/word*; F*_ (1,28) _ = 21.93, *p* < 0.001, *η_*p*_*^2^ = 0.44), but not of group (β-lactolin/placebo; *F*_ (1,28) _  = 2.19, *p* = 0.15, *η_*p*_*^2^ = 0.007) and time (week 0/week 6; *F*_ (1,28) _ = 0.065, *p* = 0.80, *η_*p*_*^2^ < 0.001). There was a significant interaction between group and condition (*F*_ (1,28) _ = 4.91, *p* = 0.035, *η_*p*_*^2^  = 0.15), but not between group and time (*F*_ (1,28) _ = 0.023, *p* = 0.89, *η_*p*_*^2^  = 0.001) or condition and time (*F*_ (1,28) _ = 0.012, *p* = 0.91, *η_*p*_*^2^ < 0.001). There was no statistically significant three-way interaction (*F*_ (1,28) _ = 0.483, *p* = 0.49, *η_*p*_*^2^ = 0.017). N400 measurements during the lexical decision task did not reveal statistically significant between-group differences in the neural activity of the frontal lobe.

### Neuropsychological tests

Table 2 shows the changes in VFT scores from baseline (week 0) to week 6. The β-lactolin group had a significantly higher number of recalled words beginning with “a” than the placebo group (*p* = 0.033). In the β-lactolin group, there was a significant increase in the number of recalled words beginning with “a” and a tendency of increase in the number of recalled words beginning with “shi” at 6 weeks from baseline (*p* = 0.005 and *p* = 0.063, respectively). There was no change in the scores in the placebo group; moreover, there were no significant between-group differences in the number of recalled words at week 0 (Table 2). There were no significant between-group differences in the changes in the MCI screen and Cognitrax scores from baseline to week 6 (Supplementary Table 1).

**Table 2:** Verbal fluency test results

	Group	Week 0	*p*	Week 6	*p*	*Δ*	*p*
Words starting with “a”’	Placebo	13.3±4.6	1.00	13.3±4.7	0.20	0.0±2.4	0.03
	β-lactolin	13.3±2.7		15.2±2.8^**^		1.9±2.2
Words starting with “shi”	Placebo	14.5±3.2	0.33	14.6±3.8	0.79	0.1±2.7	0.13
	β-lactolin	13.1±4.4		15.0±4.3		1.9±3.6
Animal names	Placebo	20.3±3.2	0.61	20.1±3.4	0.10	–0.2±1.7	0.11
	β-lactolin	21.0±4.5		22.5±4.4		1.5±3.4

Data are presented as means±standard deviations for the placebo and β-lactolin groups (*n* = 15 each group). Group differences were analyzed using unpaired *t*-test (shown as *p*-value in the table). The differences within each group between baseline and week 6 were analyzed using paired *t*-test; ^**^p < 0.01.

## DISCUSSION

To the best of our knowledge, this is the first study to evaluate the effects of β-lactolin on neural activity in the brain using EEG. Our findings suggest that supplementation with β-lactolin increased the P300 amplitude during auditory tasks and the number of recalled words in the VFT compared to the placebo group.

We observed that β-lactolin supplementation increased P300 amplitude at the Cp2 (a part of the parietal lobe) and C4 (at the area between the frontal and parietal lobe), which suggests that β-lactolin improved concentration and attention during the auditory tasks. Previous studies, using neuropsychological tests, have reported that β-lactolin supplementation improved attention including inhibitory and selective attention and executive function. It has been reported that P300 amplitude is associated with the inhibitory attention in persons with MCI [35]. In our study, the persons in the β-lactolin group had significantly higher number of recalled words in the VFT than the placebo group, which is consistent with our previous findings regarding improvements in the VFT [10]. Previous functional MRI studies have shown the VFT is associated with frontal and parietal area activation in healthy participants, which suggests that the parietal area is involved in attentional switching in the VFT [36]. Consistent with our neuropsychological test results, our EEG findings indicated that β-lactolin supplementation improved neural activity in the parietal area. The parietal and frontal lobes complementarily contribute to the attentional control and processing [37, 38]. Taken together, our findings suggest that supplementation with β-lactolin induces neural activation in the parietal area, which results in improved attention, executive function, and memory retrieval, the characteristics associated with the frontal lobe function. In contrast, β-lactolin supplementation did not alter the N400 amplitude during lexical tasks in healthy adults. The P300, but not N400, amplitudes and latencies have been reported to be significantly altered in the healthy older participants than in the young participants [39]. Since we evaluated the brain activity in the healthy old participants, β-lactolin supplementation enhanced the P300, but not N400, amplitude. We could not determine the effects of β-lactolin on N400. Future studies targeting patients with MCI or dementia are warranted.

We previously reported that oral β-lactolin administration activated dopaminergic neurons and increased dopamine levels in the cortex and hippocampus regions, which resulted in improved cognitive function in a rodent model [6, 40]. Positron emission tomography studies have shown dopamine as a crucial component for frontal and parietal lobe-associated attention [41, 42]. A recent randomized trials showed that supplementation with β-lactolin increased the regional cerebral blood flow in dorsolateral prefrontal cortex, and was also evident in VFT [10, 43, 44]. This suggests that the activation of monoamine system by β-lactolin in the frontal-parietal areas is associated with improved cognitive functions, including attention, executive function, and memory retrieval. NCI from Cognitrax in the current study did not show difference between the groups because the score at baseline was higher than that reported in the previous studies and the change after 6 weeks of intervention was much less [45].

The P300 amplitude is decreased in patients with MCI [46], Alzheimer’s disease [18, 47], and Huntington’s disease [48]. Reduced P300 amplitude is associated with impaired attention. P300 amplitude of auditory ERPs in patients with Alzheimer’s disease has been reported to be reduced, along with the decrease in levels of dopamine (homovanillic acid) and serotonin (5-hydroxyindoleacetic acid) metabolites. We previously reported reduced dopamine levels in the cortex region of 5×FAD mice, a model for Alzheimer’s disease. β-lactolin treatment increased dopamine levels, reduced amyloid-β levels, and improved memory impairment [9]. Moreover, supplementation with β-lactolin improved memory retrieval and attention associated with dorsolateral frontal cortex function in older healthy adults [10, 11]. This indicates that β-lactolin improves the monoamine levels and P300 amplitude, and therefore, attenuates cognitive decline and dementia.

This study has several limitations. First, we used a relatively small sample size (*N* = 15 per group), which might have affected both P300 and N400 measurements. Therefore, further studies with larger sample sizes are required to appropriately elucidate the underlying mechanism. There was an increase in P300 amplitude in the auditory, but not visual task by the supplementation with β-lactolin. Therefore, we should evaluate the effects of β-lactolin on tasks other than the auditory tasks, including previously reported cognitive functions, which were improved by β-lactolin supplementation, to elucidate the general effects of β-lactolin on the parietal area. No significant between-group difference was observed in N400 measurement during the language tasks. Moreover, the present study did not evaluate the washout effects of β-lactolin in cognitive function tests and ERP, and serum levels of β-lactolin at several time points. As mentioned above, future studies with older adults having cognitive decline, including MCI and dementia, are required to evaluate the improvements in N400 component with β-lactolin supplementation.

In conclusion, to the best of our knowledge, this is the first study to show that supplementation with β-lactolin increases neural activity, as indicated by the P300 amplitude, in the parietal area during auditory tasks requiring attention. Therefore, supplementation with β-lactolin may be an effective approach for enhancing cognitive function.

## Supplementary Material

Supplementary Material

## Data Availability

The datasets used and/or analyzed during the current study are available from the corresponding author on reasonable request. The supplementary material is available in the electronic version of this article: http://dx.doi.org/10.3233/JAD-201413.
