# Supplementary material for: β-Lactolin Enhances Neural Activity, Indicated by Event-Related P300 Amplitude, in Healthy Adults: A Randomized Controlled Trial
Source: J Alzheimers Dis. 2021 May 18;81(2):787–96. doi: 10.3233/JAD-201413 (PMC8203246; doi:10.3233/JAD-201413)
Supplement: Supplementary Material [file jad-81-jad201413-s001.pdf]

# Supplementary Material

**β-Lactolin Enhances Neural Activity, Indicated by Event-Related P300 Amplitude, in Healthy Adults: A Randomized Controlled Trial**

**Supplementary Table 1.** Changes in mild cognitive impairment (MCI) screen and Cognitrax scores

| | **Placebo** | **β-lactolin** | ***p*** |
|---|---|---|---|
| MCI screen (MPI) | 3.6 ± 5.6** | 3.3 ± 7.4 | 0.903 |
| Cognitrax (NCI) | 1.5 ± 5.2 | 0.6 ± 4.1 | 0.625 |

Data are presented as means ± standard deviations for placebo and β-lactolin groups (n = 15 each group). Group differences were analyzed using unpaired *t*-test. The differences between baseline and week 6 were analyzed using paired *t*-test; ***p* < 0.01. MPI, memory performance index; NCI, neurocognition index
